# Bioprocess development of 2, 3-butanediol production using agro-industrial residues

**DOI:** 10.1007/s00449-022-02761-5

**Published:** 2022-08-12

**Authors:** Sulfath Hakkim Hazeena, Narasinha J. Shurpali, Henri Siljanen, Reijo Lappalainen, Puthiyamdam Anoop, Velayudhanpillai Prasannakumari Adarsh, Raveendran Sindhu, Ashok Pandey, Parameswaran Binod

**Affiliations:** 1 Microbial Processes and Technology Division, CSIR-National Institute for Interdisciplinary Science and Technology (CSIR-NIIST), Thiruvananthapuram, 695019 Kerala India; 2 Academy of Scientific and Innovative Research (AcSIR), Ghaziabad, 201002 India; 3 Department of Environmental and Biological Sciences, University of Eastern Finland, Kuopio campus, Kuopio, Finland; 4 Natural Resources Institute Finland (Luke), Halolantie 31 A, 71750 Maaninka, FI Finland; 5 Biomaterials Technology, Dept. of Applied Physics & SIB-Labs, University of Eastern Finland (Kuopio Campus), Yliopistonranta 1 F, 70211 Kuopio, FI Finland; 6 Centre for Innovation and Translational Research, CSIR-Indian Institute of Toxicology Research, Lucknow, 226 001 India; 7 Sustainability Cluster, School of Engineering, University of Petroleum and Energy Studies, 248 007, Dehradun, India; 8 Centre for Energy and Environmental Sustainability, Lucknow, 226 029 India

**Keywords:** 2, 3-Butanediol, Biomass, Fermentation, Bioprocess

## Abstract

The valorization of agricultural and industrial wastes for fuel and chemical production benefits environmental sustainability. 2, 3-Butanediol (2,3-BDO) is a value-added platform chemical covering many industrial applications. Since the global market is increasing drastically, production rates have to increase. In order to replace the current petroleum-based 2,3-BDO production, renewable feedstock's ability has been studied for the past few decades. This study aims to find an improved bioprocess for producing 2,3-BDO from agricultural and industrial residues, consequently resulting in a low CO_2_ emission bioprocess. For this, screening of 13 different biomass samples for hydrolyzable sugars has been done. Alkali pretreatment has been performed with the processed biomass and enzyme hydrolysis performed using commercial cellulase. Among all biomass hydrolysate oat hull and spruce bark biomass could produce the maximum amount of total reducing sugars. Later oat hull and spruce bark biomass with maximum hydrolyzable sugars have been selected for submerged fermentation studies using *Enterobacter cloacae* SG1. After fermentation, 37.59 and 26.74 g/L of 2,3-BDO was obtained with oat hull and spruce bark biomass, respectively. The compositional analysis of each step of biomass processing has been performed and changes in each component have been evaluated. The compositional analysis has revealed that biomass composition has changed significantly after pretreatment and hydrolysis leading to a remarkable release of sugars which can be utilized by bacteria for 2,3-BDO production. The results have been found to be promising, showing the potential of waste biomass residues as a low-cost raw material for 2,3-BDO production and thus a new lead in an efficient waste management approach for less CO_2_ emission.

**Graphical Abstract:**

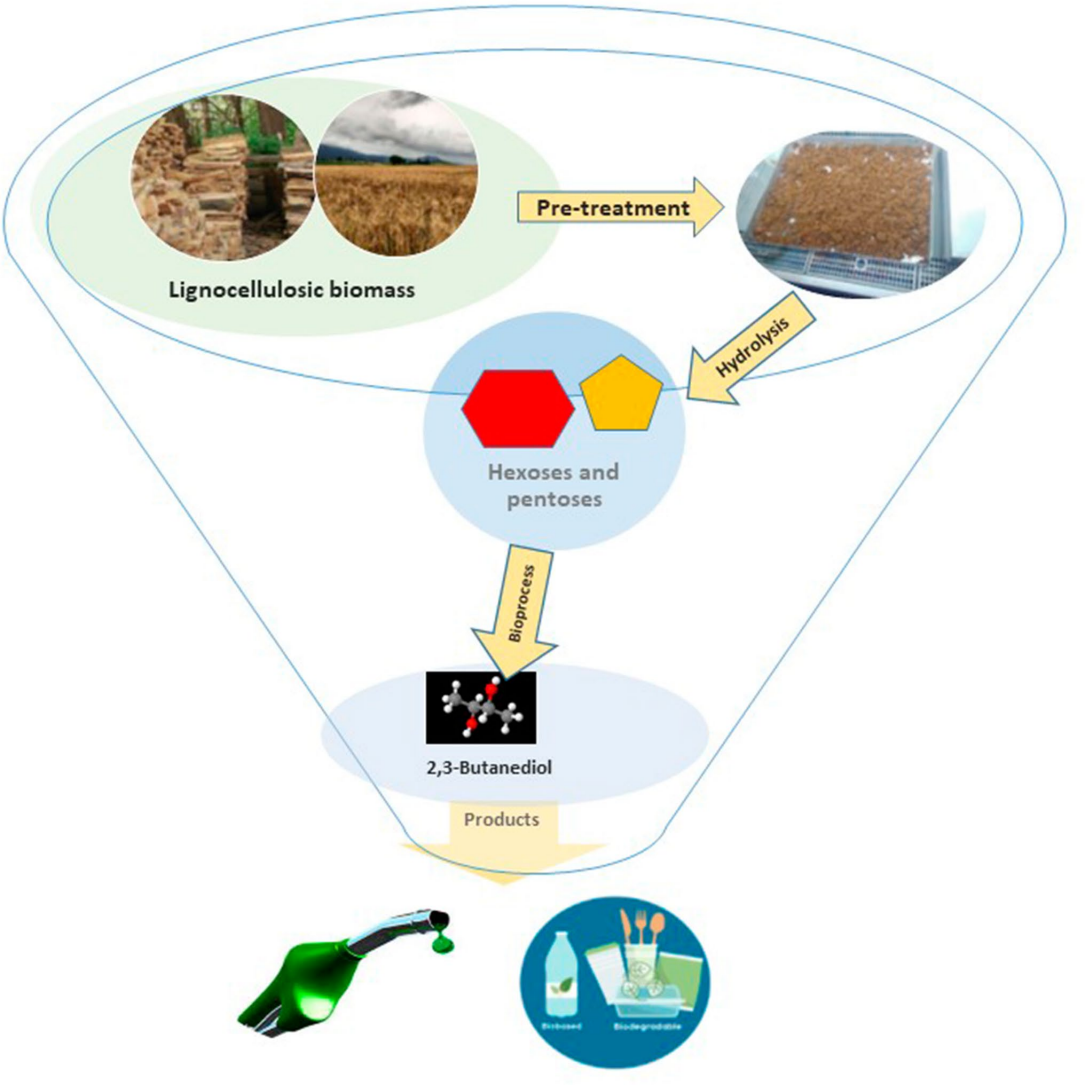

## Highlights

- 13 different agro-industrial residues were tested for fermentable sugars.
- Oat hull and spruce bark biomass hydrolysate were used for 2,3-Butanediol production.
- Batch fermentation with oat hull hydrolyzate yielded 37.59 g/L of 2,3-butanediol.
- Fermentation using spruce bark hydrolyzate yielded 26.74/L of 2,3-butanediol.

## Introduction

Despite current global regulations for climate change, atmospheric greenhouse gas levels are skyrocketing. According to the International Energy Agency (IEA), global energy-related CO_2_ emission was around 33 gigatonnes in 2019 (IEA (2020). This number reveals that our regulatory approaches are not impressive enough to cope with real-world emissions. Climate-related risks are adversely affecting health, food security, water supply, and thereby economic growth. More effective strategies and policies have to be shaped to tackle this issue precisely.

Dependence on fossil fuel is the major cause of anthropogenic emissions, not only for fuel and power but also for the manufacture of platform chemicals. Many value-added platform chemicals that are currently being synthesized via petrochemical routes can be derived from renewable biomass. 2, 3-Butanediol (2,3-BDO) is currently produced from petroleum routes and potentially could be produced from biomass [1].

2, 3-BDO has wide applications in agriculture, pharmaceuticals, and polymer industry. It can be derivatized to high-value fuel additive due to its high heat of combustion [2]. Optically pure 2,3-BDO is used in the synthesis of chiral compounds [3]. Levo (2R,3R (–)) form of 2,3-BDO has been used as a potential antifreeze agent in the pharmaceutical industry because of its very low freezing point (− 60 °C) [4]. According to Ameco market research, 2,3-BDO market is expected to reach 10300 million US dollars by 2025.

Biological synthesis of 2, 3-BDO has a long history from 1906. It was first reported in *Klebsiella pneumoniae* and later in other species such as *Klebsiella*, *Serratia*, *Enterobacter*, *Bacillus*, and *Paenibacillus* *polymyxa* [5]. Along with these native producers, a couple of heterologous hosts such as *S. cerevisiae* and *E. coli* were successfully demonstrated to produce 2, 3-BDO [6] [7]. Different renewable feedstock such as ligncellulosic biomass, biodiesel-derived glycerol and non-crop plants were tested for 2, 3-BDO production. Studies shows that sugarcane bagasse pretreated with green liquor, containing Na_2_CO_3_ and Na_2_SO_3_, followed by enzymatic hydrolysis can be used as a carbon source for producing 2,3-butanediol. The yield of 0.395 g/g sugar was reached after 72 h of fermentation, indicating that the lignocellulosic biomass could be used to produce 2,3-BDO affordably using metabolically modified *Enterobacter aerogenes* [8]. Saratale and coworkers successfully performed the pretreatment of kenaf core biomass with inorganic salts and calcium peroxide along with their use in the synthesis of 2,3-BDO [9]. Biologically derived 2, 3-BDO was commercialized by a few industries [5].

Current biological production yields are not sufficient enough with wild-type strains. Substrate cost is another major limiting factor in industrial bioprocess for 2, 3-BDO production. Its production from agro-industrial residues of the Finnish ecosystem has relevance in terms of waste management system for less CO_2_ emission. Integrating a waste management system for value-added chemical production will have potential benefits. The current study covers a wide spectrum of biomass wastes from Finnish agricultural and industrial sector and evaluates their ability for generating fermentable sugars. Later the study demonstrates a renewable methodology for production of 2,3-BDO from biomass hydrolysate via fermentation. To decrease the current anthropogenic emission, an attempt has been made by adopting a renewable route for the production of 2,3-BDO, and in future for the commercial synthesis of 2,3-BDO this will be a relevant reference. The study aims to develop a renewable route for the production of 2,3-BDO using agricultural waste from Finnish agricultural sector. For this purpose, different agricultural residues were chosen and different pretreatment strategies were employed for the degradation of cellulose structure. Later, enzymatic hydrolysis of cellulose was performed using commercial cellulase and the resulting hydrolysate has been used for 2,3-BDO fermentation. Among all biomass, one with highest release of total reducing sugars has been chosen for further experiment. *Enterobacter cloacae* SG1 has been used for 2,3-BDO fermentation and the efficiency of the process evaluated. Moreover, the compositional analysis of biomass after each step of pretreatment, hydrolysis, and fermentation was performed to evaluate the effectiveness of the process.

## Materials and methods

### Media and chemicals

**(**2S, 3S)–( +), (2R, 3R)–( −)-and meso-2,3BDO and acetoin (> 98%) were procured from Merck (Germany). All other chemicals of analytical grade were used in this study. Fermentation media components were (in gram per Liters) yeast extracts-5.0, KH_2_PO_4_-6.0, K_2_HPO_4_-14.0, Sodium citrate dehydrate-1.0, ammonium sulphate-2.0, and magnesium sulphate heptahydrate-0.2.

### Biomass samples

Thirteen agro-industrial residues were selected for the study. The agro-residual biomass samples and biogas digestate were received from Maaninka Research Station, Kuopio, Finland. Leaf samples were collected from Municipal Sewage Waste collection facility, Kuopio, Finland. Wood bark and chip samples were local industrial samples used for bioenergy production. Hemp hurd was obtained from Futura 75 fiber hemp grown in Northern Savo. Paper mill effluent samples were received as frozen from Mondi Powerflute Oy, Kuopio, Finland. The raw biomass, except oat hull and barley hull, were milled to a particle size of 3–5 cm length, 2–3 cm breadth and 1 cm thickness, dried and stored at room temperature until used. Oat hull and barley hull were processed for pretreatment and hydrolysis as such from industrial residues normally used for bioenergy.

### Pretreatment of biomass

The biomass samples (15% w/w) were pretreated in 250 mL Erlenmeyer flasks with 1.5% NaOH (w/w) at 121 °C, 15 lbs for 20 min. After cooling, excess alkali was washed thoroughly with water and filtered and dried at 65 °C for 12 h and subjected to enzymatic hydrolysis. Liquid samples such as paper mill effluents and biogas digestate were tested for reducing sugar availability without any treatment.

### Enzymatic hydrolysis

Enzyme hydrolysis of biomass was performed in 1 M citrate buffer (pH 4.8) using *Trichoderma reesei* cellulase (Sigma Aldrich). The hydrolysis conditions were as follows: biomass loading 10% (w/w), enzyme loading 20 FPU (filter paper unit) and antibiotic loading 0.001% (w/w) incubation at 50 °C, 200 rpm. Water and buffer were added to the biomass and allowed to equilibrate at 50 °C. After this, the antibiotic solution and enzyme were added so that the fermentation reaction could proceed. Samples were collected in every 24 h and checked for total reducing sugars.

### Preliminary screening of the biomass for sugar yield

For the preliminary screening of the biomass materials, the pretreated and hydrolysed samples were analyzed for reducing sugars by DNS method [9].

### Compositional analysis

The composition of selected native and alkali pretreated samples were determined according to National Renewable Energy Laboratory (NREL) analytical methods for biomass (Sluiter et al. 2011).

### Culture maintenance

*Enterobacter cloacae* sp.SG1 was maintained as glycerol stocks and sub cultured regularly in nutrient agar plates. Peptone, beef extract**** and NaCl were used for preparing seed media.

### Fermentation for 2, 3-BDO production

Fermentation medium was inoculated with 24 h-old inoculums. Media components and biomass hydrolysate were sterilized by autoclaving separately and added during the time of inoculation. 20 g/L purified glucose was added to the media separately. Fermentation conditions were pH 6.5, temperature at 37 °C, and 200 rpm for 120 h. Samples were withdrawn periodically and analyzed for 2, 3-BDO and sugar [10].

### Analytical methods

Total reducing sugar concentration was estimated by DNS method [11]. Compositional analysis of biomass at various experimental stages was done according to NREL protocol [12]. Bacterial growth was monitored spectrophotometrically by checking optical density at 620 nm (Shimadzu, Japan).

### Statistical analysis

The experiments were repeated for a minimum of three times. All data were expressed as means ± SD. Statistical differences between control and treated groups were evaluated using Student’s t test, and differences between groups were considered statistically significant at *p*-values < 0.05.

## Results

### Preliminary screening of the biomass

Preliminary screening of agricultural and industrial biomass for 2, 3-BDO fermentation was done based on the ability to release reducing sugars for microbial growth and fermentation. For this purpose, alkali pretreatment was done at high temperature and pressure which would eventually lead to lignin removal and disrupt the crystallinity of cellulose. Then enzymatic hydrolysis of cellulose was performed using cellulase from *Trichoderma reesei*. Cellulase enzyme cleaves cellulose fibers to individual glucose molecules and finally, this can be utilized by bacteria for growth and 2, 3-BDO production. Upon alkali pretreatment and hydrolysis, the sugar release from the biomass at 24 h of hydrolysis was estimated (Table 1).

**Table 1:** Total reducing sugar concentration in different biomass

Sl No	Biomass	Total reducing sugars (g/L) at 24 h of hydrolysis
1	Hemp hurd	46.36 ± 7.65
2	Aspen bark	20.93 ± 0.03
3	Oat hull	84.27 ± 0.09
4	Barley	22.57 ± 0.30
5	Spruce bark	46.21 ± 0.92
6	Wood chips	22.9 ± 0.00
7	Birch bark	0.00
8	Leaf type A	46.8 ± 2.8
9	Leaf type B	27.49 ± 8.8
10	Digestate sample 1	1.72
11	Digestate sample 2	2.04
12	Paper mill condensate	Nil
13	Wood chip wash water	1.71 ± 0.98

Among all biomass tested except paper industry wastes such as wood chip wash water and paper mill condensate possess a little amount of reducing sugars in it. Biogas digestate samples also contain a small amounts of free sugars. Birch bark biomass was also detected with zero amount of reducing sugars. All other biomass including leaf wastes, hemp, oat, barley and aspen biomass contains a significant amount of sugars that can be valorized for energy purposes or can be used as the low-cost feedstock for fermentation. Maximum sugar release during 24 h of hydrolysis was found from oat husk (84.27 g/L) followed by hemp (46.36 g/L) and spruce bark biomass (46.21 g/L). The sugars released from leaf samples were also found to be promising. The liquid samples such as paper mill effluent and biogas digestate contain very little or no reducing sugars. Oat hull and spruce bark biomass which gave a higher amount of reducing sugars were selected for 2,3-BDO fermentation.

### Compositional variability of biomass

The composition of native, alkali-pretreated biomass and the residue after enzymatic hydrolysis are presented in Table 2. The composition analysis provides detailed picture of the native composition of individual components in biomass and the changes that occurred in each step of pretreatment and hydrolysis. Composition analysis thus assists in calculating the efficacy of the whole process from pretreatment to fermentation.

**Table 2:** Compositional variation of the biomass used in the study

Sample description	Cellulose (%)	Hemicellulose (%)	Lignin (%)	Ash (%)	Total (%)
Oat hull biomass native	61.52 ± 0.95	7.31 ± 0.37	22.24 ± 0.58	1.53 ± 0.15	92.6 ± 1.93
Oat hull biomass pretreated	64.59 ± 1.78	11.19 ± 1.21	12.7 ± 1.87	0.399 ± 0.033	88.88 ± 2.54
Oat hull biomass residue after hydrolysis	31.92 ± 1.2	19.68 ± 1.03	12.98 ± 0.3	0.42 ± 0.011	64.57 ± 22.41
Spruce bark biomass native	47.15 ± 1.64	11.80 ± 0.43	44.55 ± 0.26	0.166 ± 0.033	103.67 ± 2.06
Spruce bark biomass pretreated	64.96 ± 0.28	6.78 ± 1.07	29.75 ± 1.02	0.099 ± 0.033	101.59 ± 2.34
Spruce bark biomass residue after hydrolysis	34.06 ± 0.92	13.17 ± 1.34	25.32 ± 0.44	0.37 ± 0.003	72.49 ± 0.79

The cellulose content in the native oat hull was higher (61.52) than that of spruce bark (47.15). After alkali pretreatment, hemicellulose fraction was found to increase in its percentage, mainly because of the lignin removal. Lignin removal was efficient in oat hull and close to 50% lignin removal was observed in alkali-pretreated biomass (12.7) compared to the native (22.24). The lignin content in spruce (44.55) is almost twofold higher than oat hull (22.24). A notable amount of lignin was reduced in spruce biomass (from 44.55 to 29.75) in pretreatment. A corresponding increase was observed with cellulose fraction (47.15 to 64.96) in spruce biomass at the same time. In enzymatic hydrolysis 33% of cellulose was hydrolyzed in oat hull while it was 31% in spruce bark. After enzyme hydrolysis, lignin fraction was reduced from 22.24% to 12.98% in oat hull biomass while it is 44.5 and 25.32, respectively, in spruce. The relative removal of lignin concentration was prominent in spruce biomass.

### Pretreatment and enzymatic hydrolysis

The cellulose in biomass ws degraded to glucose and finally, utilized by the bacteria for growth and 2, 3-BDO production. DNS analysis of oat hull and spruce bark biomass showed 31.56 g/L and 25.45 g/L of total reducing sugars, respectively. Detailed HPLC analysis revealed individual sugars present in hydrolysate. 11.32, and 9.62 g/L glucose was present in oat hull and spruce bark biomass hydrolysate, respectively. Along with glucose 2.22 and 4.01 g/L of cellobiose was found in oat hull and spruce bark hydrolysate fraction. Additionally, arabinose (2.6 g/L) was also found present in oat hull hydrolysate. The hydrolysate was a mixture of both pentoses and hexoses with slight amount of inhibitors such as acetate.

### Fermentation of biomass hydrolysate for 2, 3-BDO production

**Fig.1:**
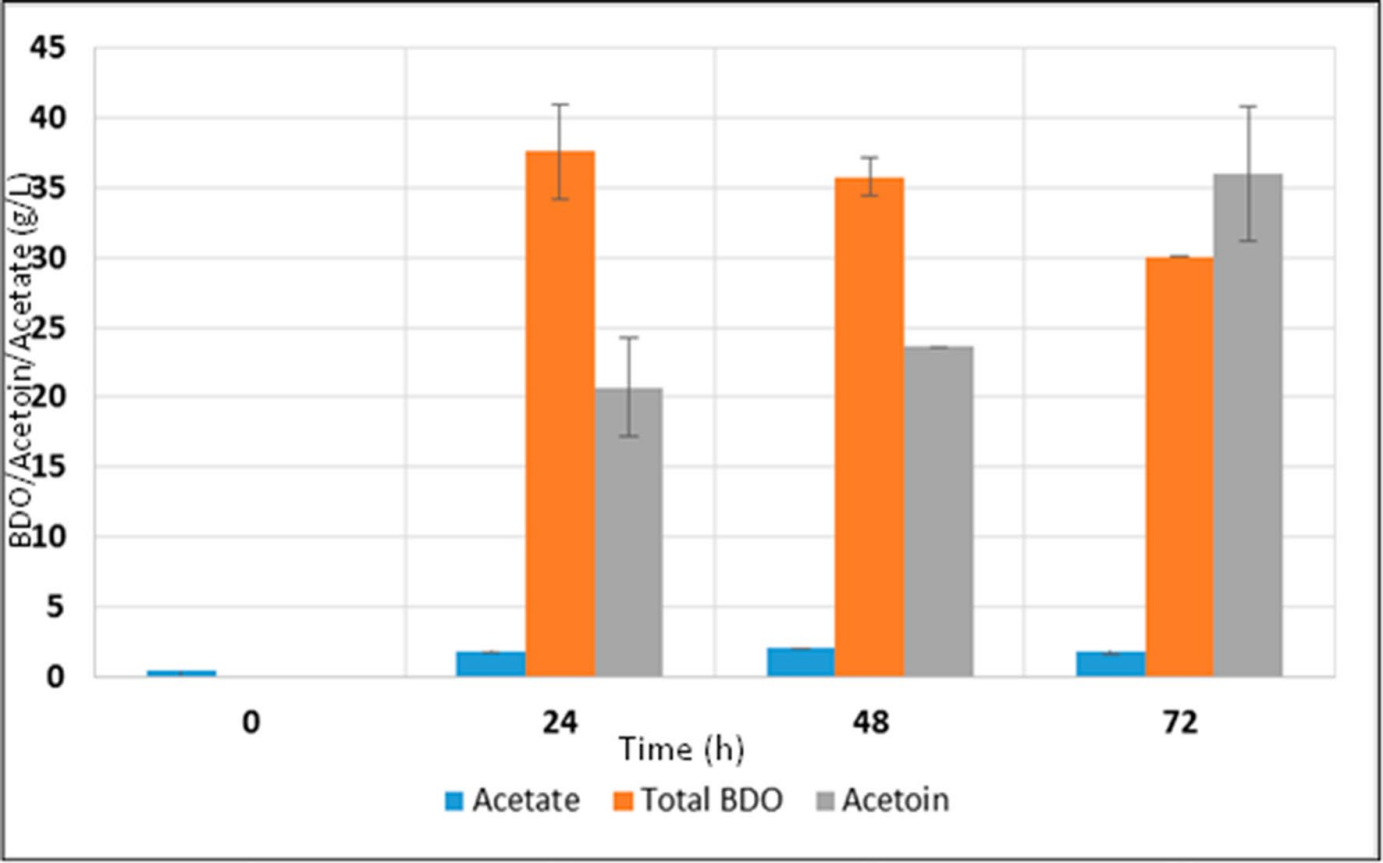
Production of 2,3-BDO using oat hull hydrolysate

**Fig.2:**
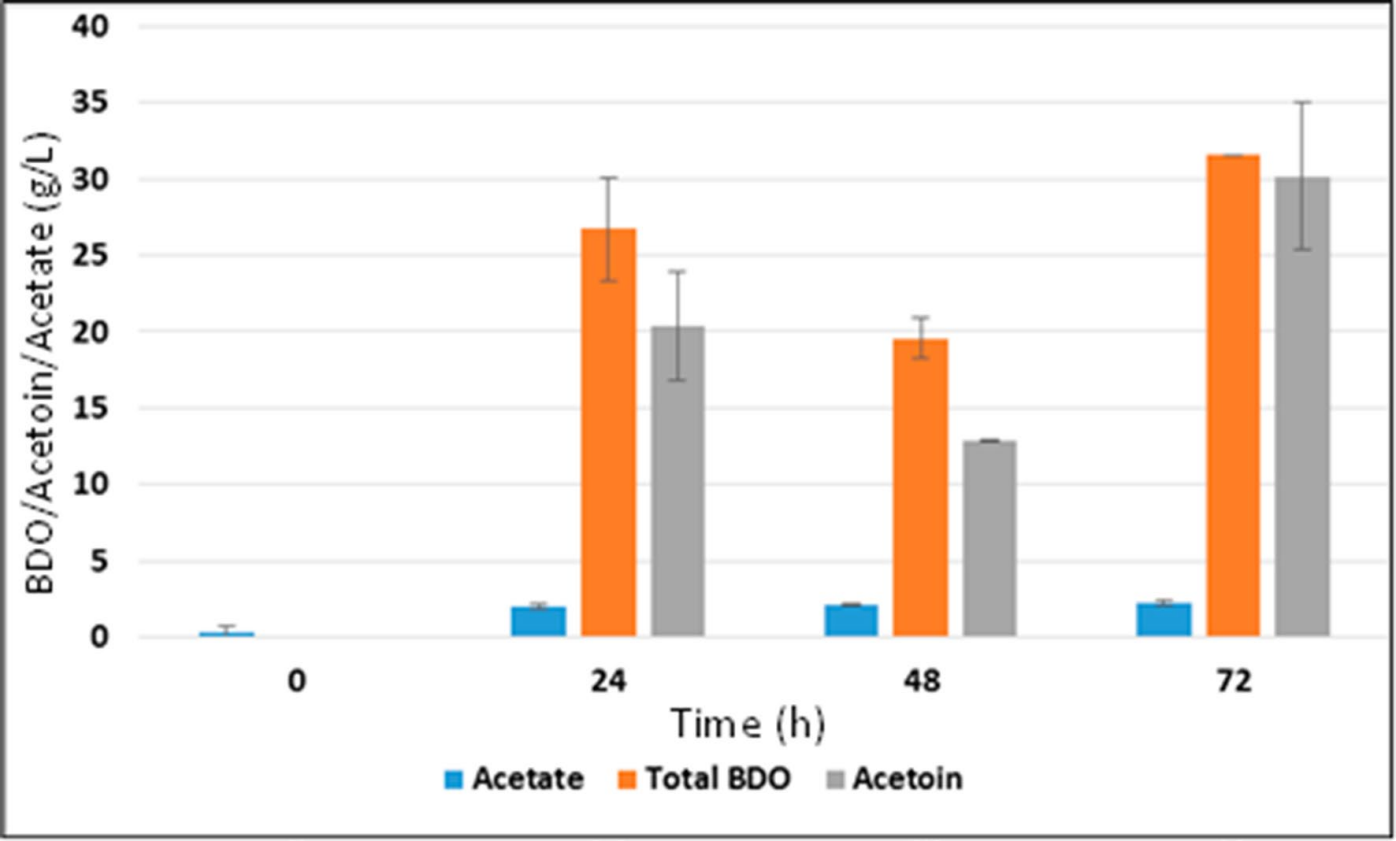
Production of 2,3-BDO using spruce bark biomass hydrolysate

2, 3-BDO fermentation was initiated by the inoculation of *Enterobacter cloacae* SG1 into the hydrolysate medium. HPLC analysis reveals the individual concentration of major products during 2, 3-BDO fermentation. Apart from 2, 3-BDO, acetate and acetoin were produced predominantly and their concentrations depicted in Figs. 1 and 2. After 24 h of each batch fermentation, 37.59 g/L of 2,3-BDO from oat hull hydrolysate were obtained. Also, 20.72 g/L of acetoin was found to be co-produced with 2, 3-BDO (Fig. 1). Around 26.74 g/L of 2,3-BDO was produced from spruce bark biomass along with 20.36 g/L of acetoin (Fig. 2). Since the reaction between 2, 3-BDO and acetoin is reversible it is clear from the figure that in 24 h, 2, 3-BDO concentration decreases while that of acetoin increases. The direction of reaction could be changed depending upon the concentration of each of the involved products. When the medium became more anaerobic there was a tendency to accumulate acetoin while 2,3-BDO prefers microaerophilic conditions. This can be noted by the accumulation of acetoin along the time of fermentation. A gradual decrease in the oxygen level in the medium would result in acetoin accumulation. The maximum 2,3-BDO production was achieved in 24 h of fermentation and almost 90% sugar utilization was also noticed within this period. Even though acetoin concentration was found to be increasing after 24 h, the maximum 2,3-BDO was obtained at 24^th^ hour itself. So prolonging the fermentation time does not have any remarkable effect on product yield. In oat hydrolysate, 0.39 g/L of acetate was present initially after pretreatment. Acetate is formed in the hydrolysate mainly because of the hydrolysis of acetyl groups in hemicellulose [13]. Acetate is one of the major products of mixed acid fermentation pathway. Acetate concentration gradually increased and reached a maximum of 2.0 g/L in 48 h of fermentation.

### Growth of *E. cloacae* SG1 in biomass hydrolysate

**Fig.3:**
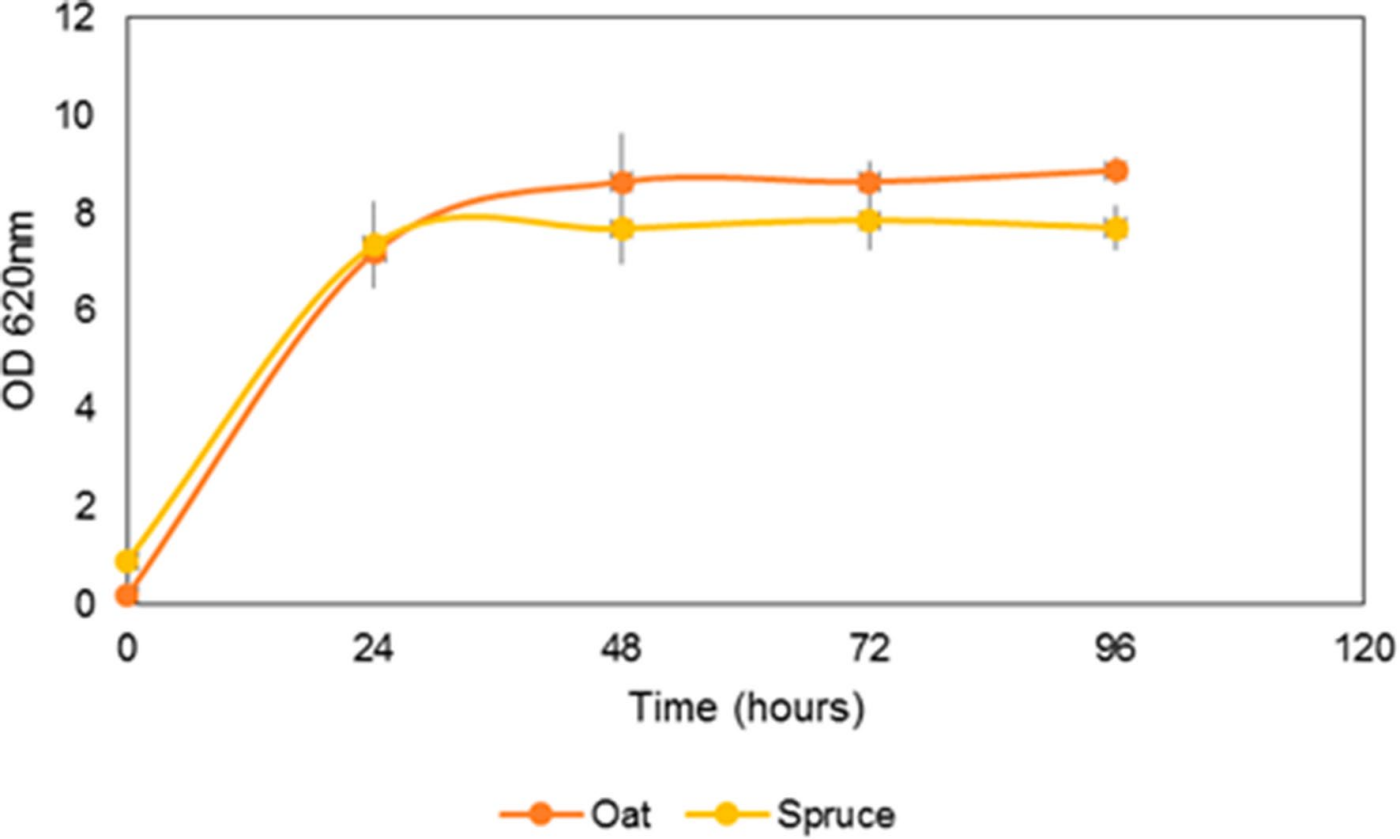
Growth pattern of *E. cloacae* SG1 in oat hull and spruce bark hydrolysate

Growth of *E. cloacae* SG1 was monitored spectrophotometrically in each 24 h of fermentation. The growth pattern of *E. cloacae* SG1 in Fig. 3 was a prototype of bacterial growth curve. The logarithmic phase was achieved at 24 h of incubation and later it proceeded to the stationary phase. Growth in spruce biomass hydrolysate was slightly retarded compared to oat hull biomass. This may be because of the presence of pigments and phenolic compounds present in the bark.

## Discussion

2,3-BDO fermentation using different low-cost substrates, both lignocellulosic and non-lignocellulosic feedstock, has been well studied in the past decades. The substrates include food industry wastes, wood hydrolysate, algal biomass, molasses, and many other non-crop substrates like Jerusalem artichoke tubers [14] [15] [16] have successfully been evaluated for its ability to produce 2,3-BDO. This study attempted to valorize a broad-spectrum agricultural and industrial waste for 2,3-BDO production especially from Finnish agricultural and industrial area. This comprehensive evaluation of agricultural waste residues, particularly for the Finnish agricultural sector, was reported for the first time. All the biomass tested were available abundantly and at cheap costs. Since one of the challenges in bioenergy production is substrate cost [17] and availability, these factors should be considered essential criteria for selecting feedstock.

Almost a major number of biomasses tested contain a significantly higher amount of reducing sugars. However, the splendid availability of oats hull and spruce bark biomass raises them for special attention. The oat cultivation status of Finland for the past years showed a relative abundance of oat availability. According to Luke (Natural resources Institute, Finland), 1200 million kilograms of oat were cultivated in Finland in 2019. Accordingly, a significant amount of oat hull has been generated after its processing. Burning up this much biomass will bring environmental concerns as we mentioned in the introduction. Oat hull comprises 28% of grain weight and contains 45% cellulose [18]. Among the biomass, oat and spruce bark biomass have been found to be the maximum reducible sugars and were selected for further studies. Even though studies explaining the cellulose composition of different biomass including spruce [19] and oat [20] the composition can vary due to different factors including species variation and climatic conditions [21].

Composition analyses have revealed the amount of each component of the lignocellulose biomass. Lignin can act as hindering the binding of cellulose degrading enzyme by contributing non-specific binding [22]. It can also act as a potential source of origin of phenolic compounds that eventually leads to retardation of bacterial growth [23]. A significant amount of lignin could be removed by alkali pretreatment [24]. Alternate pretreatment strategies such as alkaline and organic solvent treatment on biomass such as corn stover, poplar and Douglas fir produced significant amount of sugars and on subsequent fermentation of the sugars resulted 2,3-BDO [25]. 2,3-BDO production has also been reported using Jatropha hulls after ionic liquid pretreatment followed by dilute acid hydrolysis [26]. Similarly, in oat and spruce biomass, after efficient lignin removal, enzymatic hydrolysis has been reported to lead to  an efficient conversion of polysaccharides to monosaccharides. 

As the spruce bark biomass was composed of pigments and phenolics, and the hydrolysate was itself darker in appearance. Because of such inhibitory compounds, a retarded growth, and corresponding less 2,3-BDO production were noted with spruce biomass hydrolysate compared to oat hull. Similar observation was noted with Strizincova et al., in spruce bark biomass [27]. Phenolic compounds and degradation products of sugars are inhibitory compounds for microbial growth and fermentation. The inhibitory phenolic compounds are generally removed by overliming [28]. Here no treatment for inhibitor removal was performed because it will cause significant removal of sugars and reduce the final product yield. In the case of oat hull biomass, even though lignin removal was happening to a good extent, cellulose fraction was not increasing accordingly. While analyzing the sugar composition of oat biomass hydrolysate along with glucose, arabinose was also found to be present. The strain *E.cloacae* SG1 is known for its ability of using both hexoses and pentoses [29]. While using biomass-derived sugars it is important to check the organism’s ability to utilize different sugars as the carbon source for fermentation. Recently a newly isolated *Cronobacter sakazakii* was reported for its ability to utilize both glucose and xylose for 2,3-BDO production [30].

Other than monosaccharides a smaller amount of cellobiose was also found to be present in the hydrolysate. This indicates the incomplete hydrolysis reaction that occurred in cellulose fraction. The incomplete hydrolysis can reduce the efficiency of fermentation as the bacteria cannot use partially hydrolyzed cellulose [31]. The effectiveness of hydrolysis of biomass has to be addressed in an industrial bioprocess. While using oat and spruce biomass hydrolysate significant amount of 2,3-BDO was produced. Okonkwo and coworkers observed similar 2,3-BDO production from non-detoxified wheat straw hydrolysate using *Paenibacillus polymyxa* DSM 365 [32]. Similarly, 32.7 g/L 2,3-BDO was produced from nondetoxified sugarcane bagasse hydrolysate-derived sugars by *Enterobacter ludwigii* [33]*. *It has been demonstrated that, while conducting a non-sterile fermentation using non-sterile food waste using a thermophilic Bacillus licheniformis YNP5-TSU, the 2,3-BDO production was less in comparison with sterile conditions [34]. An improvised study using the same bacteria showed a significant increase in 2,3-BDO production because of the increase in initial sugar concentration in the hydrolysate [35]. It showed the effect of hydrolysis and media components on the diol titers. Different biomass and the corresponding 2,3-BDO titers using microbial fermentation are depicted in Table 3. Other than this biomasses, Brewers’ spent grain hydrolysate, bakery waste, and bread waste have also been found as potential source for 2,3-BDO production [36] [37] [38].

**Table 3:** Biomass hydrolysis for 2,3-BDO fermentation

Biomass	Pretreatment if any	Microorganism	2,3-BDO (g/L)	Reference
Non-detoxified wheat straw	Dilute acid hydrolysis	*Paenibacillus polymyxa* DSM 365	32.0	[32]
Jatropha hull	Ionic liquor pretreatment	*Klebsiella oxytoca*	33.49	[26]
Sugarcane bagasse	Hydrothermal pretreatment	*Enterobacter ludwigii*	32.7	[33]
Non sterile food waste	NA	*B. licheniformis* YNP5-TSU	5.9	[34]
Food waste hydrolysate	NA	B. licheniformis YNP5-TSU	36.7	[35]
Oat hull Spruce bark	Alkali pretreatment	*Enterobacter cloacae* SG1	37.59 26.74	This study

2,3-BDO can be recovered from fermentation broth by aqueous two phase extraction system using an organic solvent. Aqueous two phase extraction has been successfully used in the separation of 2,3-BDO produced using biomass hydrolysate [39]. The results were promising within a scale-up possibility since the 2,3-BDO titers using oat hull and spruce bark biomass were optimal. Valorizing lignocellulose waste material could be beneficial by reducing the CO_2_ emission and utilizing the reserved carbon as fuel and chemicals [40]. Other than the final product yield, efficiency of pretreatment and hydrolysis reactions, byproduct accumulation and the energy-mass balances have to be considered for the upgradation of the process. The capital costs, operating expenses, mass balances, and energy balances all can be estimated using the technoeconomic analysis method, which is based on experimental data [41]. A solution for managing agricultural and industrial waste, a significant solid waste in Finland, is provided by the circular economy strategy illustrated in this paper, which also produces large yields of industrially important 2,3-BDO.

## Conclusion

The biomass samples from Finnish agricultural and industrial sector were tested for 2,3-BDO production of fermentable sugars. Among the 13 biomass samples tested, oat hull, spruce bark biomass, hemp hurd, and leaf waste produced significant amount of fermentable sugars (84.27, 46.21, 46.36, and 46.8 g/L, respectively) and these biomass were found to be promising low-cost substrates for valorization to fuel and chemicals. Alkali pretreatment followed by enzyme hydrolysis significantly alters the composition of biomass by releasing the sugar moiety from cellulose fraction which could finally be used in 2,3-BDO fermentation. Results on 2,3-BDO fermentation was found to be promising and have the potential to be upscaled to industrial level. 2,3-BDO fermentation with oat hull and spruce bark biomass could produce 37.59 and 26.74 g/L, respectively, in submerged fermentation. The successful production of high-value 2,3-BDO from comparably cheaper biomass helps in developing efficient strategies for commercialization of biomass-derived fuels and chemicals. Increasing our knowledge about unexploited biomass wastes for value-added chemicals and fuels will have a bright future in renewable energy generation.
